# Effects of Alpha Transcranial Alternating Current Stimulation on Stress Reactivity and Decision Making: A Randomized Sham-Controlled Trial

**DOI:** 10.1016/j.bpsgos.2026.100731

**Published:** 2026-04-09

**Authors:** Philippe Vignaud, Emmanuel Poulet, Lilas Robert, Véronique Raverot, Nathalie Prieto, William Vallet, Jerome Brunelin

**Affiliations:** a Le Vinatier, Psychiatrie Universitaire Lyon Métropole, Bron, France; b PsyR2 Team, Centre de Recherche en Neurosciences de Lyon U1028 UMR5292, Université Claude Bernard Lyon 1, Centre National de la Recherche Scientifique, Institut National de la Santé et de la Recherche Médicale, Bron, France; c Centre Régional du Psychotraumatisme Auvergne-Rhône-Alpes, Hôpital Edouard Herriot, Hospices Civils de Lyon, Lyon, France; d Psychiatric Emergency Service, Hospices Civils de Lyon, Lyon, France; e Laboratoire de Biologie Médicale Multi-Sites, Centre de Biologie et de Pathologie Est, Groupement Hospitalier Est, Hospices Civils de Lyon, Lyon, France; f WAKING Team, Centre de Recherche en Neurosciences de Lyon U1028, Université Claude Bernad Lyon 1, Centre National de la Recherche Scientifique, Institut National de la Santé et de la Recherche Médicale, Bron, France

**Keywords:** Decision making, Delay discounting, DLPFC, Stress, tACS

## Abstract

**Background:**

Evidence suggests that alpha-band power plays a crucial role in stress response and decision making (DM). Transcranial alternating current stimulation (tACS) is a noninvasive brain stimulation technique capable of modulating brain oscillations in a frequency-specific manner. In this study, we investigated whether alpha tACS (α-tACS), delivered to healthy individuals while they experienced an acute stress situation, would influence biological stress responses and DM performance.

**Methods:**

In a double-blind, sham-controlled study, 38 healthy individuals were randomly assigned to receive either α-tACS (2 mA, 30 min, 10 Hz; *n* = 19) or sham stimulation (*n* = 19) on the prefrontal cortex (PFC) during stress exposure. Salivary cortisol reactivity was measured repeatedly to assess the biological stress response, DM was assessed with the delay discounting task.

**Results:**

Compared with sham (mean area under the curve with respect to ground [AUCg] = 76.4, SD = 29.63), α-tACS (mean AUCg = 103.4, SD = 49.08) significantly increased stress-induced cortisol release (*p* = .048, Cohen’s *d* = 0.342). α-tACS also significantly decreased discounting rates following stress whereas no effect was observed in the sham condition.

**Conclusions:**

Although some limitations, such as the absence of electroencephalography recordings and high variability in outcomes should be considered, these findings suggest that modulation of prefrontal alpha oscillations via tACS may reduce top-down regulatory control from the PFC, resulting in an enhanced biological stress response. This effect was accompanied by alterations in DM processes, supporting a functional relationship between frontal alpha activity, stress reactivity, and DM. These mechanisms offer promising therapeutic prospects for stress-related disorders like posttraumatic stress disorder, which involve altered alpha-band oscillations and blunted cortisol reactivity.

## Plain Language Summary

Stimulating prefrontal alpha brain activity increased the release of the stress hormone cortisol compared with sham stimulation in acute stress situation. The same stimulation reduced impulsive decision making. These findings suggest that modulating prefrontal alpha activity may link how the brain biologically responds to stress with how decisions are made under stressful conditions.

Stress is a universal experience, essential for adaptation to environmental challenges. The response to acute stress is mediated by several physiological pathways, most notably the hypothalamic-pituitary-adrenal (HPA) axis. Activation of the HPA axis involves a series of feedforward and feedback mechanisms that result in the release of cortisol by the adrenal glands. Cortisol plays a key role in mobilizing energy, modulating inflammation, and maintaining homeostasis during stressful situations.

At the brain level, the prefrontal cortex (PFC), which plays a key role in regulating the response to stress (1,2) through its high density of glucocorticoid receptors (GRs) (3,4), is also critically involved in high order cognitive functions (5, 6, 7, 8). When exposed to acute stress, a decrease in PFC activity is observed, accompanied by a shift in cognitive state toward heightened alertness. More specifically, several functions linked to PFC activity, such as cognitive flexibility, decision making (DM), and working memory, are affected by stress (9, 10, 11). DM is a fundamental cognitive process of human behavior by which an option is selected among a set of alternatives based on subjective preferences. DM may be affected by stress as well: even if the type of DM task remains a notable moderating factor, a tendency to choose immediate rewards was reported, especially in the economic DM tasks (12,13), and a correlation was reported between a lower cortisol release and a higher appetence for immediate rewards (14) as assessed with a subtype of DM task, the so-called delay discounting task (DDT) (15). Moreover, delay discounts seem decreased in patients with psychiatric diseases where exposure to an extreme stress factor is a core feature as observed for instance in posttraumatic stress disorder (PTSD) (16,17).

The key role of the PFC, and particularly the dorsolateral PFC (DLPFC), in regulating the stress response is further supported by noninvasive brain stimulation (NIBS) studies. These studies have consistently shown that applying excitatory stimulation protocols to the PFC, especially the left PFC, can reduce stress-induced cortisol release, both in healthy individuals and clinical populations (18). Specifically, a single session of transcranial direct current stimulation (tDCS) applied over the DLPFC (anode over the left coupled with cathode over the right) during acute stress has been reported to both decrease cortisol release and prevent stress-induced impairments in DM in healthy volunteers (19,20) and at-risk populations (21). However, the mechanisms by which DLPFC tDCS modulates both HPA axis reactivity and higher-order cognitive functioning in acute stress situations remain unclear.

Electroencephalography (EEG) has been widely used to investigate the neural correlates of the stress response. A recent meta-analysis investigated whether exposure to acute stress affects spectral band power and found that stress significantly reduces alpha-band power (22). Moreover, alterations in alpha oscillation in the frontal cortex during stress exposure have been associated with cortisol reactivity (23,24). The alpha band, typically ranging from 8 to 12 Hz, is the dominant neural oscillation frequency observed in healthy awake individuals at rest (25,26). Evidence suggests that alpha oscillations have an inhibitory function and play an active role in information processing, including DM (27). Transcranial alternating current stimulation (tACS) is a form of transcranial electrical stimulation, alongside other techniques such as tDCS, which is one of the most widely studied. tACS delivers an alternating sinusoidal current through 2 electrodes placed over the scalp of a participant, in which voltage oscillates between positive and negative values every half cycle (28). The principle behind tACS is to entrain neural oscillations by mimicking the brain’s natural rhythmic activity (29). Supporting this, studies have shown that a single session of tACS applied at alpha frequency (i.e., α-tACS) can modulate alpha-band activity in targeted brain regions (30), including the PFC (31, 32, 33).

Taken together, converging evidence suggests a close relationship between the DLPFC, alpha-band activity, and stress response. Given the ability of tACS to selectively modulate specific electrophysiological oscillations, we hypothesized that a single session of tACS delivered at the alpha frequency over the DLPFC would influence stress response. Specifically, because alpha power typically decreases during exposure to acute stress, we hypothesized that externally driven alpha entrainment would inhibit the top-down control predominantly mediated by the DLPFC, thereby leading to an increased cortisol release. The aim of the present study was to evaluate the effects of a single α-tACS session applied over the PFC on HPA reactivity and DM performance in healthy participants exposed to an acute stress situation.

## Methods and Materials

### Sample

In a double-blind, randomized, sham-controlled trial, 40 healthy adult participants (male and female, right handed), ages 18 to 45 years, were enrolled. Based on previous findings (20), a total sample size of 40 participants was estimated to be sufficient to detect a 25% difference in stress-induced cortisol release between the active and sham groups, assuming a statistical power of 80%. They were randomly allocated (using 1:1 randomization by blocks) to receive either active α-tACS (*n* = 20) or sham stimulation (*n* = 20). To control for potential effects of the menstrual cycle on cortisol reactivity (34), only female participants using hormonal contraception were included. Use of hormonal contraception was verified twice through clinical examination. The current prescription was required to be presented prior to inclusion. Exclusion criteria were as follows: 1) personal or first-degree familial current or past psychiatric disorders (DSM-5); 2) regular intake of psychopharmacological medication or beta-blockers; 3) recent exposure to a traumatic event; or 4) pregnancy. Further demographic characteristics of the sample are presented in Table 1.

### Ethical Approval and Registration

The study was approved by the local ethics committee (Comité de Protection des personnes, Ile de France I, Paris, France, on September 8, 2023) and by the French National Agency for the Safety of Medicines and Health Products (2023-A01368-37). All participants provided written informed consent after receiving a complete explanation of the study procedures. The study was preregistered in a public database (ClinicalTrials.gov identifier: NCT06229002), first posted on January 29, 2024.

### Stimulation Procedure

Stimulation was delivered using a neuroConn DC Stimulator (neuroConn GmbH). Electrodes were placed over the left and the right DLPFC, corresponding to the F3 and F4 locations according to the International 10-20 EEG electrode placement system. The current was delivered through two 3 × 3 cm electrodes. The stimulation consisted of α-tACS applied at 10 Hz for 30 minutes, with a 30-second fade-in and fade-out period, and a peak-to-peak intensity of 2 mA (±1 mA).

The sham stimulation consisted of delivering 1 minute of active stimulation using the same settings and electrode montage, including a 30-second fade-in and fade-out period, followed by no current for the remaining 29 minutes of the total 30-minute session. Blinding was ensured using the device’s Study Mode, which delivers active or sham stimulation based on a coded input unknown to both the administrator and participant. Codes were assigned by a third party, keeping participants, experimenters, and statisticians blinded. To ensure transparency and future study replication, the Report Approval for Transcranial Electrical Stimulation (RATES) checklist is included in Table S1, according to Nejati *et al.* (35).

### Stress Paradigm

To minimize nychthemeral effects of cortisol levels and reduce interindividual variability, all experimental sessions were conducted in the morning, starting at 8:30 am. Stress was induced using a modified version of the Maastricht Acute Stress Test (MAST) (36). As previously described (20,21), the MAST combines alternating phases of physical and cognitive stress of varying duration. Physical stress consisted of repeated hand immersion trials in cold water (8 °C), while cognitive stress involved mental arithmetic calculation (e.g., counting backward from 2043 in steps of 17 as quickly and accurately as possible, aloud in front of the experimenters). The stress task began at 9:50 am, lasted for 10 minutes, and was preceded by a 5-minute preparation/anticipation phase.

**Figure 1:**
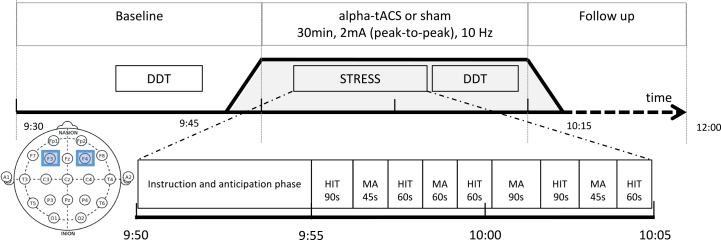
Illustration of the study design. The stress task alternates between hand immersion trial (HIT) (in water 8 °C) and mental arithmetic (MA). F3 and F4 indicate positions of the electrodes according to the International 10-20 electroencephalography electrode placement system corresponding to the prefrontal cortex. For cortisol reactivity samples, baseline was calculated from 4 samples collected before 9:45 am. The transcranial alternating current stimulation (tACS) (10 Hz) session began at 9:50 am. During the stimulation/stress procedure, samples were collected every 5 minutes at 9:55 am, 10:00 am, and 10:05 am. After the stress procedure, additional samples were collected every 15 minutes at 10:20 am, 10:35 am, and 10:50 am. DDT, delay discounting task.

Concomitant with stress exposure, participants received either active α-tACS or sham stimulation. The MAST task began 5 minutes after the start of the stimulation. Details of the experimental design are provided in Figure 1.

To control for potential effects of sleep quality (37) and nicotine (38), the participants were instructed to refrain from nicotine or alcohol intake, as well as from activities that could disrupt sleep quality (e.g., exercise and parties) on the evening preceding the experiment.

### Measures of Stress Reactivity

The primary outcome was cortisol secretion, measured by repeated salivary cortisol samples. Saliva was collected using Salivette. To assess the kinetics of cortisol release, saliva samples were collected 10 times throughout the study: 4 baseline samples taken approximately 10 minutes apart before the stress paradigm; 3 samples collected every 5 minutes immediately after the stress paradigm; and 3 additional samples collected every 15 minutes thereafter (see Figure 1). The Salivettes were centrifuged at 2000*g* for 3 minutes and stored at −20 °C until analysis by liquid chromatography–tandem mass spectrometry (LC-MS/MS) (unit of assessment: nmol/L) relative to reference values, which is considered as a gold standard for this assay. In addition, subjective stress levels were assessed using a visual analog scale (VAS) ranging from 0 to 10.

### Delay Discounting Task

DM capacities were assessed twice: once before and once after the stress/stimulation procedure using the DDT (15). The DDT evaluates individual preferences by presenting choices between smaller immediate rewards and larger delayed rewards, thereby measuring impulsivity and reward seeking in intertemporal DM and have been associated with DLPFC activity (39). Participants completed a total of 144 hypothetical trials in which the amount and delay of rewards were systematically varied. For example, one item might ask, “Would you prefer $20 today or $200 in 40 days?”

Discount rates from the DDT were calculated using a hyperbolic discounting model of the form:

(1) $$V=A\;/\;\left(1+kD\right)$$

where V represents the subjective value of the delayed reward, A is the amount of the delayed reward, D is the delay in days, and *k* is the individual’s discount rate, reflecting the degree to which future rewards are devalued. A higher *k* value indicates steeper discounting of delayed rewards, reflecting more impulsive DM. Because the distribution of *k* values is typically right skewed, a logarithmic transformation [log(*k*)] was applied before statistical analysis (39).

### Statistical Analyses

Analyses were performed using JASP software (version 0.18.3). Baseline sociodemographic characteristics were compared using Student’s *t* test for continuous variables and Fisher’s exact test for categorical variables.

The primary analysis of stress reactivity compared the active and sham groups using the Student *t* test on the kinetics of cortisol release, assessed by the area under the curve with respect to ground (AUCg) and area under the curve with respect to increase (AUCi), calculated according to Pruessner *et al.* (40). The AUCg reflects the total cortisol output by the HPA axis and serves as an index of the overall intensity of the stress response. In contrast, the AUCi captures hormonal changes over time and is considered as more specific indicator of HPA axis sensitivity to the stressor (41).

As secondary analyses, repeated-measures analyses of variance (ANOVAs) were undertaken to investigate main effects of time and group and interaction for cortisol and DDT measures. Subjective stress levels between groups and secondary outcomes, including safety, were compared using Student’s *t* tests.

## Results

Among the 40 enrolled participants, 2 were excluded from the analysis: one withdrew consent before participating in the experimental phase, and one was excluded due to issues with cortisol assay reliability. There were no significant differences between the α-tACS and sham groups in sociodemographic characteristics (Table 1).

**Table 1:** Sociodemographic Characteristics of the Participants

	Sham, *n* =19	tACS, *n* =19	*p*
Age, Years	32.1 (8.7)	31.5 (8.9)	.85
Sex, Female/Male	10/9	8/11	.75
Tobacco Use, Yes/No	1/18	4/15	.34
Salivary Cortisol, nmol/L	3.82 (1.62)	3.75 (1.75)	.79

Values are presented as mean (SD) or *n*.

Student’s *t* tests were used for quantitative intergroup comparisons. Fisher’s exact tests were used for proportion comparisons. Participants were instructed to refrain from tobacco use starting the evening before the experimental day.

tACS, transcranial alternating current stimulation.

**Table 2:** Questionnaire of Sensation Related to Stimulation

	Sham	tACS	*p*_uncorrected_	*p*_FDR_
Pain	1.05 (0.23)	1.32 (0.67)	.11	.242
Fatigue	1.68 (0.82)	2.32 (0.67)	.01^a^	.055
Burning Sensation	1.21 (0.54)	1.21 (0.63)	.99	.99
Itching	1.11 (0.32)	1.26 (0.56)	.29	.403
Tingling	1.16 (0.37)	1.32 (0.58)	.33	.403
Warmth/Heat	1.16 (0.37)	1.21 (0.54)	.73	.803
Metallic/Iron Taste	1.00 (0.00)	1.11 (0.46)	.32	.403
Phosphenes	1.00 (0.00)	1.32 (0.67)	.04^a^	.147
Altered Cognition	1.16 (0.37)	1.42 (0.69)	.15	.275
Difficulty Concentrating	1.42 (0.61)	2.00 (0.82)	.01^a^	.055
Acute Mood Changes	1.00 (0.00)	1.21 (0.54)	.09	.242

Values are expressed as mean (SD). Student’s *t* tests were used for intergroup comparisons.

FDR, false discovery rate; tACS, transcranial alternating current stimulation.

a Significance, FDR (Benjamini-Hochberg).

### Effect on Cortisol Reactivity

**Figure 2:**
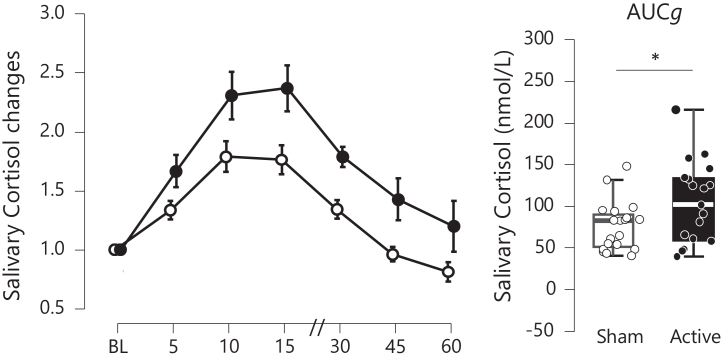
Elevation of cortisol release following stress exposure in individuals receiving either active 10-Hz transcranial alternating current stimulation (black) or sham stimulation (white). Left: Bars represent SEs. Baseline is the average of 4 measurements taken prior to stress exposure. Results are expressed as the percentage increase relative to the baseline measurement. Time is expressed in minutes after the beginning of the stress procedure. Right: Area under the curve with respect to ground (AUCg) represents the total cortisol release over time in nmol/mL. Box plots show the distribution of the variable. The central line represents the median, the box indicates the IQR (Q1–Q3), and whiskers extend to the minimum and maximum values. ∗*p* < .05.

The primary analysis revealed a significantly greater stress-induced cortisol release in the α-tACS group (mean AUCg = 103.4, SD = 49.08) compared with the sham group (mean AUCg = 76.4, SD = 29.63) (*t*_36_ = −2.047, *p* = .048, Cohen’s *d* = 0.342, 95% CI [−1.314 to −0.006]) (Figure 2, right panel). For the AUCi, a trend toward a significant difference was observed between the α-tACS group (mean AUCi = 37.3, SD = 33.78) and the sham group (mean AUCi = 22.0, SD = 24.89) (*t*_36_ = −1.588, *p* = .121, Cohen’s *d* = 0.335, 95% CI [−1.159 to 0.135]).

The repeated-measures ANOVA revealed a significant main effect of time (*F*_1,36_ = 20.0117, *p* < .001, η_p_^2^ = 0.357) and a trend toward significant main effect of group (*F*_1,36_ = 3.961, *p* = .05, η_p_^2^ = 0.099), but no significant time × group interaction (*F*_1,36_ = 0.938, *p* = .47, η_p_^2^ = 0.025). This indicates a significant increase in cortisol after the stress task across all participants, regardless of stimulation condition, in both the α-tACS and sham groups (Figure 2, left panel).

### Effects on DDT

Repeated-measure ANOVA on log(*k*) at the DDT revealed a main effect of time (*F*_1,36_ = 5.675, *p* = .023) between before and after stress, no effect of group (*F*_1,36_ = 0.035, *p* = .85), and no interaction (*F*_1,36_ = 2.860, *p* = .09). Exploratory post hoc comparisons indicated a significant decrease in discounting rate after stress in the active α-tACS group (mean difference = 1.061, SE = 0.368, *p*_Holm_ = 0.040), whereas no significant change over time was observed in the sham group (0.493, SE = 0.733, *p*_Holm_ = 1.00). Key contrast findings are provided in Table S2.

### Safety and Blinding

Overall, α-tACS was well tolerated, with no serious adverse events reported. However, participants in the α-tACS group reported higher levels of fatigue, difficulty in concentrating, and phosphenes compared with the sham group (see Table 2). However, none of these differences survived correction for multiple comparisons following false discovery rate correction. This had no effect on the blinding quality, as neither the participants nor the experimenters were able to correctly identify the stimulation condition, likely because attention was primarily focused on stress-induced emotional changes rather than on sensations related to the stimulation itself. In addition, there was no effect on subjective stress levels or on the emotional experience of the experimental procedure, as indicated by analyses of the subjective VAS scores. Stress-induced self-rated subjective stress and emotional experience did not differ significantly between groups: the active group (mean VAS score = 5.0, SD = 2.47) versus the sham group (mean VAS score = 4.1, SD = 1.9) (*p* = .22).

## Discussion

The main goal of this study was to investigate the effect of a single α-tACS session, delivered bilaterally over the PFC (more precisely on the left and right DLPFC), on cortisol reactivity following exposure to a standardized stress task.

### Effects on Cortisol Reactivity

Our results showed that the participants receiving concomitant α-tACS exhibited significantly greater stress-induced cortisol release compared with those in the sham group. A major explanation for these findings may lie in the core features of the alpha band and the impact of NIBS on alpha oscillations. First, the alpha band has been suggested to act as a trait marker in the context of acute stress exposure, typically decreasing in healthy individuals facing stressful situation (22). Importantly, alpha power is thought to be inversely correlated with cortical excitability (42, 43, 44). However, in our study, α-tACS was applied to participants during exposure to an acute stress factor, with the expected effect of increasing alpha power in the PFC, effectively counteracting the physiological reduction of alpha activity normally induced by stress. Consistent with this, our findings align with a previous study in which the participants received a single session of continuous theta burst stimulation (cTBS) over the DLPFC and showed an exacerbation of cortisol release following stress (45). This is particularly relevant to our results, as EEG data suggest that cTBS can increase alpha power in the targeted brain region (46), reflecting a mechanism similar to that proposed for α-tACS. Furthermore, it is important to consider that the PFC plays a crucial but nonlinear role in regulating the HPA axis. While frontal alpha asymmetry may exhibit heterogeneous changes under stress (22), several studies have suggested that alpha activity serves as a state marker in predicting stress reactivity (23,24). In our study, α-tACS was applied bilaterally over the PFC. Given the alternating nature of the electrical current delivered by the α-tACS, it is plausible that α-tACS altered the natural activity of the PFC in a way that diminished its regulatory control over the HPA axis. In contrast, our findings diverge from those of tDCS studies, which have reported that anodal tDCS over the left DLPFC coupled with cathodal stimulation over the right DLPFC may prevent stress-induced cortisol release in both healthy individuals (20) and at-risk populations (21). However, the effects of tDCS on alpha power remain inconclusive. While some studies suggest that anodal stimulation can increase alpha power in the stimulated brain region, cathodal stimulation appears to have little or no effect on alpha oscillations (47), thereby limiting the interpretability of tDCS-induced effects on stress-related outcomes and alpha-band oscillations.

By preventing the typical stress-induced reduction in alpha activity through α-tACS stimulation, we may have disrupted the negative feedback loop that normally restrains HPA activation, thereby leading to an increase in stress-induced cortisol release. Moreover, even if the α-tACS delivered in our work stimulated only the DLPFC directly, an indirect action of subcortical regions remains a plausible hypothesis. First, the PFC, the hippocampus, and the amygdala are core components of the so-called limbic system, which provides a top-down control over the HPA axis (48). This top-down control may supply a dense network of interactions and bilateral loops of negative feedback between the HPA, the PFC, the hypothalamus and a high density of GRs on these structures (49,50). Second, just like the other NIBS tools, several studies reported that tACS could affect some distant cerebral areas indirectly through their connectivity with the stimulated target (51,52), including dopamine transmission that is also involved in DM (53,54).

Another perspective should not be neglected. Indeed, the increased cortisol release is part of a physiological reaction, leading to a large range of adaptative modifications (55). For instance, some studies also reported a positive correlation between an increased cortisol release and improved cognitive performance (56), raising the possibility that α-tACS may have enhanced adaptive physiological responsiveness rather than exacerbating stress.

### Effects on DM

We also found that tACS modulated DM under stressful conditions. Specifically, α-tACS induced a significant decrease in discounting rates following stress, whereas no stress-related change was observed in participants from the sham group. These findings align with those previously reported (57), showing that bifrontal α-tACS can reduce impulsive DM, as reflected by lower discounting rates compared with sham stimulation, suggesting that an alpha entrainment could stabilize DM. Similarly, lower discounting rates were also reported after a single session of cTBS over the right DLPFC (58).

By contrast, there is a discrepancy between our results and some findings from tDCS studies, which suggest that reducing stress-induced cortisol release through prefrontal anodal tDCS is also associated with a decreased preference for immediate rewards on the DDT and decreased cortisol release (20). This divergence highlights a potential mechanistic difference between tACS and tDCS in modulating both stress reactivity and DM behavior under stress.

In addition, impulsivity may also increase when strong emotions are present (59,60). A recent meta-analysis found out that a single session of tDCS delivered over different subregions of the PFC induced a weak but significant decrease in emotional reactivity to a stress factor (61), so one could also hypothesize that the tACS impairs emotional control, and thereby mediate the reduced impulsivity.

Moreover, although stress is often assumed to impair cognitive abilities, its effects appear to be more nuanced. Several studies have shown that stress impairs long-term memory retrieval (62), whereas it can enhance memory encoding (63), memory retention (64), and aspects of DM (65). A meta-analysis further indicated that stress does not exert an overall main effect on inhibition, a key component of DM. Notably, within inhibition, stress was found to impair cognitive inhibition while enhancing response inhibition. In addition, by contrasting the effects of cortisol administration on executive functions with those of stress exposure, stress was shown to influence executive functioning through additional pathways beyond cortisol alone (66). Consistent with this complexity, a recent review examining the effect of cortisol on DM reported heterogeneous outcomes depending on moderating factors, such as task type and sex (12). Similarly, although many studies on alpha-rhythm reactivity have concluded that alpha power is selectively attenuated by attention-demanding mental activity, other reports have described instances in which increased attention is associated with unchanged or even enhanced alpha power—a phenomenon known as the paradoxical response (67). Importantly, very few studies have investigated the electrophysiological correlates of the DDT, and none have specifically focused on the alpha-band activity. Consequently, the interactions between stress, cortisol, DM, and alpha power are likely nonlinear. Our findings should therefore not be considered paradoxical; rather, they highlight the need for further studies to better elucidate the impact of alpha entrainment on DM processes.

### Perspectives

The main result of our study opens interesting preliminary perspectives at the clinical level. Acute stress disorder (ASD) is a psychiatric condition that may occur after exposure to a potential traumatic event (e.g., natural or industrial disaster, workplace or road accident, or any form of assault). Core symptoms of ASD include flashbacks, nightmares, avoidance of trauma-related triggers, hyperarousal, and dissociative experiences. If these symptoms persist for more than 1 month, or if they begin after the first month posttrauma, the diagnosis is more accurately classified as PTSD (68). PTSD is a debilitating condition with an estimated lifetime prevalence of approximately 3.9% in the general population (69). Although the pathophysiology of ASD and PTSD is still not fully understood, a widely accepted hypothesis suggested that they result from a maladaptive and dysregulated stress response.

This dysregulated stress response has been observed through a blunted cortisol reactivity in acute stress situation (70) and on a reduced cortisol awakening response in patients with PTSD (71). In the case of ASD, the evidence is scarcer and less consistent. However, some pilot studies that measured cortisol levels within the few hours after the traumatic event reported that decreased cortisol secretion was associated with a higher risk of developing PTSD (72,73). In a related but indirect perspective, a higher cortisol awakening response has been reported as a protective factor against PTSD symptom development 1 month after the trauma (74).

Consistent with results from the current study and implicating an abnormal stress response, several studies have investigated the alpha-band oscillations in patients with PTSD. Although findings are not entirely consistent, a decrease in alpha-band power has been repeatedly observed in this population (75, 76, 77, 78).

Combined with our findings, these results suggest a potential interest of bilateral α-tACS as an early intervention in these populations. One may hypothesize this approach could help counteract the blunted cortisol reactivity observed in PTSD and suspected in ASD by increasing alpha power in acute stress situation.

Several limitations should be acknowledged. First, the inclusion of EEG recordings would have been valuable to determine whether alpha-band power was effectively modulated following the α-tACS session and to examine potential associations between alpha power and cortisol reactivity. Second, the α-tACS session was delivered at a fixed frequency of 10 Hz for all participants. However, an increasing number of studies have employed stimulation based on the individual alpha frequency (IAF). Using a fixed frequency may have influenced the results and limited the generalizability of our findings. Although the use of IAF is becoming more common in tACS literature, evidence regarding its superiority in terms of aftereffects remains mixed (79,80). Third, as consistently reported in studies involving NIBS and/or experimental stress, marked interindividual variability in both biological and cognitive responses was observed in the present sample. In addition, the relatively small sample size may have limited the statistical power to detect more subtle effects, despite a priori sample size calculation. Additionally, the relatively narrow age range of the participants is noteworthy, given that age significantly affects the HPA axis at both central and peripheral levels (81). Although no sex-specific effects were detected, the known impact of sex on HPA axis reactivity warrants further investigation. Regarding female participants, inclusion was restricted to those using hormonal contraception, as fluctuations across the menstrual cycle can influence cortisol reactivity, which has been reported to be higher during the luteal phase (34). While this criterion allowed us to control for a potential source of variability, it may also limit the generalizability of our findings. Future studies should address this issue more effectively, for example, by stratifying female participants according to menstrual cycle phase (luteal vs. follicular). A further limitation concerns the absence of a formal assessment of blinding. Although participants completed poststimulation ratings of sensations and emotional states, we did not include a direct measure asking participants to guess or estimate the stimulation condition. This issue is particularly relevant in tACS studies, where subtle stimulation-related sensations may differ between active and sham conditions and potentially influence participants’ expectations, although this was not the case in the current study. While the comparable sensation ratings observed in the current study provide indirect support for adequate blinding, these measures do not directly determine whether participants were able to discriminate between stimulation conditions. In the broader NIBS literature, poststimulation condition guessing or graded estimates of perceived stimulation are often used to support claims of effective blinding. Such approaches, including probability estimates of receiving active stimulation or ratings of perceived stimulation intensity, may provide a more direct assessment of potential unblinding. Future tACS studies could incorporate these measures alongside sensation questionnaires to evaluate blinding integrity more comprehensively and minimize the potential contribution of expectancy effects.

Lastly, to evaluate stress reactivity, we used the AUC of cortisol release as the primary outcome. Both the AUCg and the AUCi were calculated, but only AUCg reached statistical significance. Although both measures are considered appropriate for assessing stress-induced cortisol release (41) and have been used in several studies examining the effects of a single NIBS session on cortisol reactivity (82, 83, 84), further research with larger samples is needed to determine whether AUCi and AUCg are reliably affected.

### Conclusions

The findings of the current study suggest that a single session α-tACS applied bilaterally over the PFCs in healthy participants enhances cortisol reactivity under acute stress conditions. Some methodological limitations such as the small sample size and the absence of EEG recording weaken the strength of our results. Further studies are required to investigate the underlying mechanism of this effect and to determine whether a similar modulation can be achieved in individuals with psychiatric conditions such as ASD or PTSD.

## Supplementary Material

Table S1–S3

Key Resources Table
