# Supplementary material for: Effects of Alpha Transcranial Alternating Current Stimulation on Stress Reactivity and Decision Making: A Randomized Sham-Controlled Trial
Source: Biol Psychiatry Glob Open Sci. 2026 Apr 9;6(4):100731. doi: 10.1016/j.bpsgos.2026.100731 (PMC13224353; doi:10.1016/j.bpsgos.2026.100731)
Supplement: Table S1–S3 [file mmc1.pdf]

SUPPLEMENTARY INFORMATION

Effects of Alpha-Transcranial Alternating Current Stimulation on Stress Reactivity and Decision Making

Vignaud *et al.*

Supplementary Material

Supplemental Table 1: Table of RATES (Report Approval for Transcranial Electrical Stimulation)

Supplemental Table 2: Summary table of key contrast regarding the Delay Discounting Task.

Supplemental Table 3: Summary table of key contrast regarding AUCg and AUCi.

Supplemental Table 1: Table of RATES (Report Approval for Transcranial Electrical Stimulation)

**Participant**	Sample Size (Dx), Recruitment Process, Medication		40 Healthy Subjects, recruitment through notice posting and email, no medication allowed	
	Age(yrs), Sex(F:M), Edu(yrs), Handedness(R:L:D)		31.3±8.7, 18:22, n/s, (40:0:0)	
	Hours of Sleep, Consumption of Caffeine, Nicotine, and Alcohol		n/s, no caffeine, no nicotine, no alcohol from the evening preceding the experiment	
	Participant Eligibility Criteria: Right-handed, age between 18 and 45, hormonal contraception for females. Non inclusion criteria: personal or first-degree familial current or past psychiatric disorders, regular intake of psychopharmacological medication or beta-blockers, recent exposure to a traumatic event, pregnancy			
**Stimulator**	Stimulator: neuroConn DC Stimulator (neuroConn GmbH, Ilmenau, Germany).			
	Sham Option	"Study Mode" NeuroConn	Waveform	Sinusoid
	Output Channels	n/s	Stimulator Safety Features	power cut if impedance exceeds 3 X 104 Ohms
	Current Resolution	n/s	Monitoring and Feedback	Impedance check
**Electrodes**	Positioning	International 10-20 EEG system	Inter-electrode Distance	individualized
	Shape	Square	Assembly	n/s
	Size	9 cm2 (3X3)	Contact Medium	Conductive paste
	Orientation	anteroposterior	Impedance	n/s
	Material	Biocarbon	Connector Position	up
	Number: 2			
	Montage: 2 electrodes over F3 and F4 according to EEG 10/20 system			
**Current**	Intensity (mA)	(±1 mA).	Amplitude	Peak to zero (2mA)
	Density (V/m)	n/s	Personalization	n/s
	Distribution (Method)	n/s	Duration (min)	30 min
	Frequency (Hz)	10 Hz	Ramp up/down (sec)	30/30
	Polarity	n/a	Warm-up time (min)	n/s
	Waveform	Sinusoidal	Sham Characteristics	1min active, ramp up/down 30:30
**Procedure**	Study Setting and Site	n/s	Attrition (n)	2
	Hypothesis Statement	Exploratory	Blinding Method	Double blinded
	Preregistration	Clinicaltrials: NCT06229002	Ethical Considerations	yes
	Session Duration (min)	2 hours	Safety Monitoring	yes
	Total Number of Sessions	1 session	Informed Consent Process	yes
	Session Frequency	10Hz	Conflict of Interest	no
	Concurrent Intervention	Stress procedure (MAST)	tES operator	Received a specific training course
	Randomization (Method)	yes (iws, by block no stratification)	Data Analysis Plan	Yes (pre-registred)
	Counterbalancing	n/s	Data Availability	yes
	Study Design: Randomized, double-blinded,			
	Stimulation and Assessment Task Timing: Online (salivary cortisol, DDT); Offline (salivary cortisol)			
	Inter-session Interval: n/a, single session, all session conducted on the morning, same hour			
	Data Collection Time Points: 5min, 10min, 15min, 30min, 45min, 60min after tACS start: salivary cortisol; Post (level of induced stress (VAS), Side-effect survey)			
	Baseline Assessment: salivary cortisol, DDT			
	Control Intervention: Sham control (Initial ramp up/down stimulation only)			
	Outcome Measure: AUCg, AUCi, discount rate at the DDT			

AUCg: area under the curve with respect to the ground; AUCi: area under the curve with respect to the increase; DDT delay discounting task; tACS: transcranial alternative current stimulation, VAS: Visual Analogue Scale

Supplemental Table 2: Summary table of key contrast regarding the Delay Discounting Task.
SE: Standard Error; CI: Confidence Interval

Analysis	Contrast	Mean difference	SE	95% CI	Effect size (cohen's d)	p (holm)
**DDT**	Active α-tACS (pre vs post)	1.067	0.368	[0.033, 2.089]	0.469	0.040
	Sham (pre vs post)	0.180	0.368	[-0.848,1.209]	0.080	1.000

Supplemental Table 3: Summary table of key contrast regarding AUCg and AUCi.
SE: Standard Error; CI: Confidence Interval

Independent Samples T-Test

						95% CI for Cohen's d	
	t	df	p	Cohen's d	SE Cohen's d	Lower	Upper
AUCg	-2.047	36	.048	-0.664	0.342	-1.314	-0.006
AUCi	-1.588	36	.121	-0.515	0.335	-1.159	0.135
